# Investigating facilitatory versus inhibitory effects of dynamic social and non-social cues on attention in a realistic space

**DOI:** 10.1007/s00426-021-01574-7

**Published:** 2021-08-10

**Authors:** Samantha E. A. Gregory

**Affiliations:** Aston Institute of Health and Neurodevelopment, Aston University, Birmingham, B4 7ET UK

## Abstract

This study aimed to investigate the facilitatory versus inhibitory effects of dynamic non-predictive central cues presented in a realistic environment. Realistic human-avatars initiated eye contact and then dynamically looked to the left, right or centre of a table. A moving stick served as a non-social control cue and participants localised (Experiment 1) or discriminated (Experiment 2) a contextually relevant target (teapot/teacup). The cues movement took 500 ms and stimulus onset asynchronies (SOA, 150 ms/300 ms/500 ms/1000 ms) were measured from movement initiation. Similar cuing effects were seen for the social avatar and non-social stick cue across tasks. Results showed facilitatory processes without inhibition, though there was some variation by SOA and task. This is the first time facilitatory versus inhibitory processes have been directly investigated where eye contact is initiated prior to gaze shift. These dynamic stimuli allow a better understanding of how attention might be cued in more realistic environments.

## Introduction

Joint attention, i.e. the shared focus of two individuals on an object, person, or event is an important aspect of human communication and humans generally cannot help but follow other people’s eye gaze (Frischen et al., 2007a, 2007b; Kampis & Southgate, 2020; Stephenson et al., 2021). This phenomenon, known as the gaze cuing effect is studied using an adapted Posner cuing task (e.g. Posner, 1980). Targets presented in a location looked at (valid condition) by an uninformative central cue face are reliably found to be responded to faster than targets presented in the looked away from (invalid) location. Similar responses are also found for other communicative cues such as arrows and directional words (Hommel et al., 2001; Ristic et al., 2002; Tipples, 2002, 2008). However, while these cuing effects are not unique to gaze cues, joint attention does have a unique role in human communication. Joint attention has been found to be an important process in early learning (Striano et al., 2006; Tomasello, 1988), as well as leading to sophisticated mentalising processes, whereby we make inferences about other people’s intentions, an important aspect of social interaction (Capozzi & Ristic, 2018).

Understanding the facilitatory versus inhibitory nature of social and non-social cuing effects is an important aspect of understanding the mechanisms that drive the cues effects on attention. Facilitation refers to a speeding of response to validly cued targets while inhibition refers to a slowing of response to invalidly cued targets and is measured by comparing reaction times in valid, invalid, and neutral conditions. Effects are considered purely facilitatory if reaction times are faster in the valid condition than both the neutral and invalid conditions, with no difference between the neutral and invalid conditions. Effects are considered purely inhibitory if reaction times are slower in the invalid condition than both the neutral and valid conditions, with no difference between the neutral and valid conditions. Finally, effects can show facilitation with inhibition when reaction times in the neutral condition sit between the faster valid and slower invalid conditions.

Attentional facilitation and inhibition effects can be caused by the cue acting upon attention to create an intention to act upon a stimulus that may facilitate action if the cue is valid, or cause action to be inhibited if the cue is invalid. Alternatively, non-attentional facilitation can occur due to a motor response being primed toward the cued location (Hommel, 1993, 2011), this can in turn cause non-attentional inhibition due to a cue target conflict whereby the prepared response to incongruent information provided by the cue slows response to the target (Green et al., 2013). These non-attentional effects are most likely to occur at SOAs of 300 ms or less, before attention is under volitional control (Müller & Rabbitt, 1989), i.e. when intentional processing of the cue occurs. Therefore, it is important to investigate the cues effects over a time course than includes both shorter and longer SOAs.

Different tasks can reveal different aspects of the cues’ effects. In both localisation (i.e., respond with the location of the target) and detection tasks (i.e., respond when you see the target) response is made to target presence, though in localisation tasks there is the added spatial element. Therefore, for both tasks, responses are susceptible to response priming, where the participant is primed to respond to a target in the location cued leading to facilitation effects if the cue is valid and cue target conflict effects if the cue is invalid. For discrimination tasks (i.e., respond with the identity of the target) the response is not based on its presence/location, but instead requires target processing, therefore responses are less susceptible to response priming effects. Non-attentional effects of cuing are therefore most likely to be revealed by localisation and detection tasks whereas discrimination tasks are likely to reveal if the cue influences participant attention. Previous research shows inhibition without facilitation (Green et al., 2013) using a detection task, facilitation without inhibition using discrimination, detection and localisation tasks (Friesen & Kingstone, 1998; Hietanen et al., 2008) and facilitation with inhibition using detection and localisation tasks (Hietanen, 1999; Langdon & Smith, 2005). Looking at this small sample, where only one study appears to have investigated this effect using a discrimination task, it appears that inhibition effects are only revealed for localisation and detection tasks, which have this stronger element of motor response priming. Therefore, based on the current literature, it is likely that the attentional effects of gaze cues are facilitatory, without an inhibitory element. To further test this theory, here a localisation task and discrimination task are tested using dynamic, realistic cues. This therefore increases the number of discrimination tasks tested and allows further understanding of the nature of the cuing effect on attentional processes.

While the cuing tasks used to investigate these facilitatory versus inhibitory factors to date have been diverse, all have neglected the important social factor of eye contact, i.e., looking into the eyes of another person. This is because direct gaze faces often serve as the neutral condition in these tasks. Therefore, if eye contact were also engaged in the valid and invalid conditions, the movement of the eyes into the averted position would signal the onset of the target in the shift trials only. This would slow reaction times in the neutral condition for reasons unrelated to the facilitatory versus inhibitory effects of the cue (Jonides & Mack, 1984). However, eye contact, is a highly important aspect of social communication (Emery, 2000; Kleinke, 1986). Eye contact not only signals that a social interaction is occurring, but also makes the interaction feel more pleasant (Kleinke, 1986), as well as engaging and modulating distinct social processes including mentalising (Capozzi & Ristic, 2018; Conty et al., 2016; Senju & Johnson, 2009). In gaze cuing studies eye contact can be engaged by presenting a direct gaze face before an averted gaze face, and this initiation of eye contact has been found to enhance the gaze cuing effect (Bristow et al., 2007; Xu et al., 2018). Therefore, using direct gaze as a neutral cue neglects an important aspect of realistic gaze behaviour which may impact the cues effects. In addition, it is arguable that the neutral condition is not truly neutral. Eye contact both attracts and holds attention (Senju & Hasegawa, 2005; Senju & Johnson, 2009) therefore it is possible that attention is held at centre in the neutral condition slowing attentional responses to target location in a similar way to invalid cues. Further, it is possible that the onset of the lateralised stimuli in the shift conditions creates illusory motion, whereas for the traditional neutral condition there is no such effect, meaning that the neutral condition cannot be considered equivalent to the shift conditions. Therefore, in the present study eye contact is engaged by the gaze cue in all conditions by having the gaze-cue look up at the participant prior to gaze shift and then look down to the centre in the neutral condition.

As well as neglecting eye contact, investigations of gaze cuing often use highly simplistic stimuli. For example, cues used are often disembodied heads or eyes which appear in the centre of a display, further targets often appear floating in space to the side of the cue. In recent years researchers have highlighted issues with using still photographic images or schematic drawings of faces or eyes as social cues, finding that effects may not reflect those seen in real human interaction (e.g. Risko et al., 2012, 2016). To investigate cuing in more realistic environments, some researchers have used real people sat in the room with the participant (e.g. Cole et al., 2015; Lachat et al., 2012). Here, findings reflect the computer-based head or eyes-only studies, however, using real people has its own costs, and it is more resource heavy, requiring a confederate to act as the gaze cue, further, experimental control and design flexibility are limited, reducing the ability to probe effects. Virtual avatars can serve as a flexible alternative to real humans. The stimuli can be quickly and cheaply adapted to suit the research question at hand and research shows that similar social behaviours can be found during interactions with virtual agents as are seen for real human interaction (for a review, see Bombari et al., (2015)). Therefore, here, across two studies the cuing effects of virtual human avatars are compared to a non-social control cue to assess the efficacy of such stimuli. The cues presented bridge the gap between the simplicity of traditional gaze cuing tasks and the complexity of using real humans by offering videos of avatars that can be adapted and used both in screen based (as seen here) and immersive virtual tasks. The study aims to investigate the facilitatory versus inhibitory nature of the cues’ effects, while offering realism through the engagement of eye contact, cue movement, cue embodiment and the presentation of the target task.

To engage mutual eye contact, here the virtual social cues look up to meet the participants eyes prior to making a head movement to the left, right or, in the neutral condition, down. The non-social control cue, which consists of a cylindrical stick, makes an equivalent movement, pointing up to engage the participant, before making a shift to the left, right or down. This cue movement offers an important avenue of investigation. The cues are presented as videos and the directional shift movement occurs over a period of 500 ms, though, reflecting real gaze behaviour, the eyes rapidly shift gaze direction at the start of the head movement (Hayhoe et al., 2012; Hollands et al., 2002; Imai et al., 2001). Therefore, cuing effects are investigated during and at the end of the movement. This is important because in real life joint attention scenarios there is likely to be an element of head movement and yet in traditional cuing tasks the cue is either already in position, or a direct gaze image is swapped almost instantly for an image of averted gaze. Research shows that during object tracking attention as indicated by eye gaze will shift to the tracked object’s anticipated destination (Hayhoe et al., 2012). If anticipatory effects of motion occur during the movement of a central cue, cuing effects would be expected at the early 150 ms and 300 ms SOA. However, motion is also known to capture attention (Kawahara et al., 2012), and in the presented tasks there is no advantage in anticipating the destination of these non-predictive cues. Therefore, it is possible that attention will stay with the cue until it stops, meaning cuing effects would not be seen until the later 500 ms and 1000 ms SOAs.

The aim of the presented experiments was to investigate facilitatory versus inhibitory effects of cues on target response when the gaze cue is a dynamic realistic avatar, engaging eye contact prior to the gaze shift, and the control cue is a dynamic stick. This stick cue allows the motion elements of the social gaze cue to be controlled for without the learned meaning of traditional arrow cues that may confound results (e.g. Ristic & Kingstone, 2012). Experiment 1 investigated the influence of the social avatar cue and the non-social stick cue on attention orienting in a simple localisation task. Here participants had to locate a target teacup and this simple task allowed investigation of the very basic effects of these dynamic cues during (150 ms, 300 ms SOA) and at completion (500 ms and 1000 ms SOA) of the cues motion. Experiment 2 replicated and extended this paradigm, investigating the role of task difficulty (Gregory & Jackson, 2021) and response by using a more difficult discrimination task where participants discriminated between a target teapot or teacup, meaning that response was to target identity, and not target location/presence. The use of a localisation task, where responses are mapped to target location, and a discrimination task where responses are not mapped allows assessment of the extent to which these early and later orienting responses are due to attentional effects versus motor responses. Further, the range of SOAs from 150 to 1000 ms allows assessment of early (reflexive) versus later (volitional) orienting responses (Müller & Rabbitt, 1989).

## Experiment 1. localisation

### Participants and apparatus

59 participants (31 females, 28 males, mean age 26 years, range 18–47 years) were recruited online through Prolific (prolific.co) for payment. Reliable cuing effects are found with sample sizes below 20, therefore, the study was well-powered to find effects of the cues if present. All participants reported having normal or corrected to normal vision. Ethical approval was obtained from the Aston University School of Life and Health Sciences Ethics Committee. Stimuli were presented using PsychoPy3 through Pavlovia, an online study platform that has high timing accuracy (Bridges et al., 2020). Participants used their own desktop/ laptop computers to complete the task which was hosted in a web browser. Chrome or Firefox browsers were recommended but it is unknown which were used. The study and materials can be downloaded here: https://osf.io/pt6qx/, take note of the 100 ms timing discrepancy between online and desktop-based presentation in the programmed study due to the use of java script, this is explained in detail in the notes attached to the study.

### Stimuli

#### Human avatar cue

**Fig. 1:**
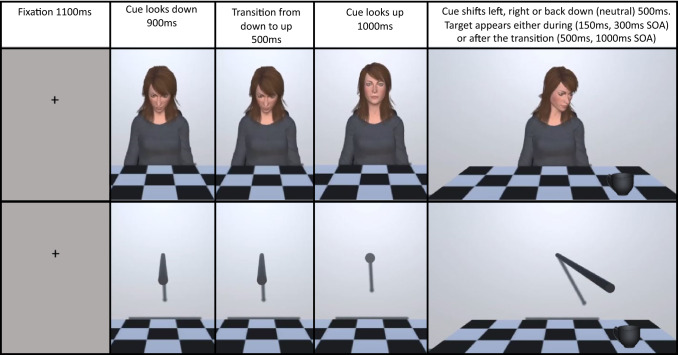
Illustration of the trial procedure and timing conditions for the two cue types: upper panel—social avatar cue, lower panel—non-social stick cue. Target shown is the teacup and both show the valid condition. Note that the trial procedure as shown is equivalent for Experiments 1 and 2, with the difference being participants response to target and the additional use of a teapot target in Experiment 2

Two male and two female identities were created showing neutral facial expressions and simple, grey clothing using Adobe Fuse (discontinued software). The avatars were uploaded to Adobe Mixamo (www.mixamo.com) where the auto rigging algorithm was used to give the avatar a movement structure and place them in a seated position. These models were then loaded into Unity where the animator was used to add looking animations (up, down, left, right). The avatars were rigid except for this head and neck movement. The avatar looked down for 900 ms (1000 ms on video, participant sees 900 ms), and then looked up by raising their head and shifting eye gaze, this transition from down to up took 500 ms for the full head movement, with the eye movement taking 30 ms. The avatar then looked at the participant for 1000 ms, engaging eye contact and then looked towards the left, right or back down to the table. This again took 500 ms, and the eye movement took 30 ms. SOAs were set from the moment that the eyes began to shift (see Fig. 1). Videos of the stimuli can be viewed and downloaded, including for use in your own research, here: https://osf.io/4zj2e/.

#### Non-social stick cue

The stick was created in Unity as a cylindrical game object which came out of the wall behind the table (see Fig. 1 and videos of the stimuli: https://osf.io/r6qb5/). This was animated using the Unity animator and movement timings matched the avatar.

#### Target

The target was a cup from the Unity asset store (White porcelain dish set demo; https://assetstore.unity.com/packages/3d/white-porcelain-dish-set-demo-82858), converted to a .png image with transparent background and rendered in grey scale. The object was rendered at 0.15 × 0.15 in height scale, therefore exact object size was dependent upon participant computer but remained to scale with the rest of the stimuli.

### Design

Within subjects’ independent variables were cue type (avatar, stick), SOA (150 ms, 300 ms, 500 ms, 1000 ms) and cue target validity (1/3 valid, 1/3 invalid, 1/3 neutral) pseudorandomised and balanced across each cue type and SOA condition. There were 12 separate conditions per cue type pseudorandomised to present 24 trials per condition. The experiment was separated into two cue type sections, within which there were two blocks of 144 trials, resulting in 288 trials per cue type. The programme randomly selected which cue type would be shown first and participants were informed of the cue type before beginning each section. The dependent variable was reaction time (RT) to correctly identify the target location.

### Procedure

To become familiar with the task, a 12-trial practice session preceded the main experiment, demonstrating each cue type and target type to the participant. Figure 1 illustrates an example trial sequence for each cue type. A trial proceeded as follows, a fixation cross was presented at the centre of the screen for 1100 ms, then replaced by the video of the cue. The cue was initially presented looking/pointing at the table, then up to the participant, and then either to the left, right or back down to the table. SOA was measured from the moment that the cue began to shift to the left, right or down. This movement took 500 ms and once finished the still image of the shifted cue remained on screen, adopting the parameters of the traditional central cuing paradigm where the cue remains on screen for the entire trial (e.g. Driver et al., 1999; Friesen & Kingstone, 1998). After the SOA period a target appeared on screen, for the 150 ms and 300 ms SOAs the target appeared while the cue continued its movement, whereas for the 500 ms and 1000 ms SOAs the target appeared when the cue had stopped. Participants were informed that the direction of the cue was not informative and should be ignored. On valid trials (1/3), the target appeared on the side towards which the cue had shifted; on invalid trials (1/3), the target appeared on the opposite side, and for the neutral trials (1/3) the target was equally likely to appear on the left or right side of the screen. The target was a teacup and was present on all trials. Participants had to localise the target as quickly and as accurately as possible using the left and right arrows on their keyboard. No specific instructions were given about which fingers or which hand to use. There was no response window cut off, but participants were told that they should try and respond more quickly if their reaction time was longer than 2000 ms. Participants received accuracy feedback on every trial and were reminded of the response keys if they were incorrect. To mitigate the greater level of distraction likely at home compared to in the lab, participants self-initiated every trial by pressing space. They were also encouraged to take breaks between blocks and between cue types.

### Data analysis

Data were analysed from correct trials only and median reaction time data was used for analysis to remove the need to eliminate reaction time outliers and control for the positively skewed nature of reaction times data (see; Jensen (1992) and Ratcliff (1993)).

Due to the uniqueness of this procedure, and to allow researchers to observe which SOAs yielded reliable cuing effects in this paradigm, a full table of results for each SOA is provided. Note that these results are uncorrected for multiple comparisons. In addition, data from all studies are accessible here: https://osf.io/5mz9j/files/.

## Results

Everyone performed at or above 97% accuracy (median = 99%). Data from incorrect trials were excluded from the reaction time analysis (< 1% of data).

A repeated measures ANOVA (multivariate) with cue type (avatar, stick), validity (valid, invalid, neutral) and SOA (150, 300, 500, 1000) as within subject factors was conducted on the median reaction times data. This showed a non-significant main effect of cue type, *F*(1, 58) = 0.206, *p* = 0.652, *ηp*^2^ = 0.004, meaning that reaction times were not statistically different between the avatar (*M* = 432 ms) and the stick cue (*M* = 430 ms).

Importantly, there was a significant main effect of validity, *F*(2, 57) = 35.709, *p* < 0.001, *ηp*^2^ = 0.556. Reaction times were significantly faster when the target was validly cued (421 ms) as compared to invalidly cued (436 ms), *t*(58) = − 7.989, *p* < 0.001, Cohen’s *d* = − 1.040 (Bonferroni corrected), further, reaction times were significantly faster when the target was validly cued as compared to when the cue stayed central (neutral condition, 434 ms) *t*(58) = − 6.957, *p* < 0.001, Cohen’s *d* = − 0.906 (Bonferroni corrected) and finally, there was no significant difference between reaction times when the target was invalidly cued as compared to neutral *t*(58) = 1.032, *p* = 0.912, Cohen’s *d* = 0.134 (Bonferroni corrected).

**Fig. 2:**
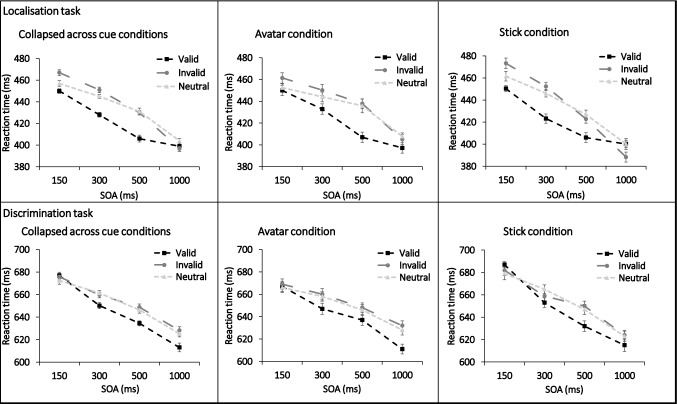
Results from Experiment 1 (upper panel) and Experiment 2 (lower panel). Reaction times are plotted for the valid, invalid and neutral condition at each SOA for the cues combined (first panel) as well as for the avatar (panel 2) and stick cue (panel 3) conditions separately. Error bars show within subjects standard error (Cousineau, 2005). See Table 1 (Experiment 1) and Table 2 (Experiment 2) for individual values and *t* test results

This validity main effect was not modulated by cue type with no significant interaction between cue type and validity, *F*(2, 57) = 0.612, *p* = 0.546, *ηp*^2^ = 0.021. However, it was modulated by SOA, with a significant interaction between SOA and validity, *F*(6, 53) = 7.983, *p* < 0.001, *ηp*^2^ = 0.475. Cuing effects were seen at the 150 ms, 300 ms and 500 ms SOAs, and not at the 1000 ms SOAs, see Table 1 for a full breakdown of effects, and Fig. 2 to visualise the differences. At the 150 ms SOA (across cues) reaction times were significantly faster in the valid condition compared to the invalid conditions (*p* < 0.001, Bonferroni corrected), though not for the valid compared to neutral condition (Bonferroni corrected *p* = 0.528), however, reaction times were significantly faster in the neutral compared to the invalid condition (Bonferroni corrected *p* = 0.036). At the 300 ms SOA, reaction times were significantly faster in the valid condition compared to the invalid condition (*p* < 0.001, Bonferroni corrected) and in the valid compared to the neutral condition (*p* < 0.001, Bonferroni corrected), reaction times were not significantly different in the neutral compared to the invalid condition (Bonferroni corrected *p* = 0.396). At the 500 ms SOA, reaction times were significantly faster in the valid condition compared to the invalid condition, (*p* < 0.001, Bonferroni corrected) and in the valid compared to the neutral condition (*p* < 0.001, Bonferroni corrected) again, reaction times were not significantly different in the neutral compared to the invalid condition (Bonferroni corrected *p* = 1). Finally, for the 1000 ms SOA, the reaction times were not significantly different between the valid and invalid conditions (*p* = 1, Bonferroni corrected) nor between the valid and neutral (*p* = 0.1, Bonferroni corrected) or invalid compared to neutral (Bonferroni corrected *p* = 0.06).

**Table 1:** Median reaction times (RTs, ms) and paired sample *t* test results (*t* value, *p* value) for the cues combined, and each cue at each SOA for the localisation task (*df* = 58), all *p* values are uncorrected for multiple comparisons and are provided for reference only, asterisks denote those that would remain significant if corrections were made

Cue	SOA	RT valid (SD)	RT invalid (SD)	RT neutral (SD)	Valid vs invalid *t* (*p*)	Valid vs neutral *t* (*p*)	Neutral vs invalid *t* (*p*)
Collapsed across cues	150	450 (125)	467 (132)	457 (123)	− 5.85 (< 0.001)*	− 2.06 (0.044)	− 3.12 (0.003)*
	300	428 (128)	451 (126)	445 (125)	− 9.11 (< .001)*	− 6.02 (< 0.001)*	− 2.18 (0.033)
	500	406 (124)	430 (125)	431 (130)	− 5.49 (< 0.001)*	− 5.90 (< 0.001)*	0.26 (0.795)
	1000	399 (113)	397 (123)	404 (115)	0.47 (0.644)	− 1.54 (0.129)	2.36 (0.005)
Avatar	150	450 (134)	461 (134)	453 (129)	− 2.91 (0.005)	− 0.55 (0.586)	− 2.01 (0.049)
	300	433 (138)	450 (133)	444 (134)	− 5.13 (< 0.001)*	− 2.65 (0.01)	− 1.39 (0.171)
	500	407 (130)	438 (134)	436 (141)	− 4.86 (< 0.001)*	− 3.95 (< 0.001)*	− 0.34 (0.739)
	1000	397 (115)	405 (130)	408 (117)	− 1.40 (0.168)	− 2.11 (0.039)	0.64 (0.524)
Stick	150	450 (123)	473 (137)	462 (121)	− 5.00 (< 0.001)*	− 2.78 (0.007)	− 2.28 (0.026)
	300	423 (125)	452 (124)	446 (122)	− 7.22 (< 0.001)*	− 7.05 (< 0.001)*	− 2.13 (0.037)
	500	406 (122)	423 (122)	427 (127)	− 4.10 (< 0.001)*	− 5.59 (< 0.001)*	1.01 (0.318)
	1000	400 (117)	388 (122)	400 (116)	2.56 (0.013)	− 0.04 (0.971)	2.91 (0.005)

There was also a significant interaction between cue type, validity and SOA, *F*(6, 53) = 4.062, *p* = 0.002, *ηp*^2^ = 0.315. Meaning that some of the differences outlined above are driven by the cue type, however, to understand this interaction requires 24 *t* tests to compare the reaction times for each cue at each SOA, resulting in a large family wise error. Instead of reporting all the tests, Table 1 shows the uncorrected *t* test results, with asterisks to denote which would remain significant if corrected using the Bonferroni method, you can also see Fig. 2 for an overview of the effects.

There was a significant main effect of SOA *F*(3, 56) = 93.596, *p* < 0.001, *ηp*^2^ = 0.834. This is due to an effect often seen in cuing studies where reaction times get faster as the SOA increases. There was also a significant interaction between cue type and SOA, *F*(3, 56) = 5.689, *p* = 0.002, *ηp*^2^ = 0.234. This is a complex effect to dig into, due to the large number of comparisons possible, importantly, comparing like for like (i.e. stick cue at the 150 ms SOA with the gaze cue at the 150 ms SOA) no comparisons are significantly different (all ps = 1, Bonferroni corrected), instead this appears to be driven by similar factors to the main effect of SOA.

### Order and gender effects

Cue order was randomised, 22 participants saw the gaze cue first, while 37 saw the stick cue first. There was no main effect of cue order (*p* = 0.151) or interaction between cue order and any condition (*p* ≥ 0.212). There was also no main effect of participant gender (*p* = 0.209) or interaction between participant gender and any condition (ps ≥ 0.074).

## Interim discussion

In Experiment 1 the influence of the cues was tested using a simple localisation task. For both cue types, reaction times were overall faster in the valid than both the invalid and the neutral condition with this being seen in all but the 1000 ms SOA. For the 1000 ms SOA, while the social avatar cue showed almost no effect on attention, the non-social cue appeared to show a flipped cuing effect, reflecting a phenomenon known as inhibition of return (Klein, 2000). This result will be returned to in the main discussion.

The key facilitatory versus inhibitory effects of the cues are dependent upon SOA. At the shortest SOA (150 ms) results appear to show inhibition, though when looking at the raw results without Bonferroni correction, this appears to reflect facilitation with inhibition, as reaction times for the neutral cue sit between the valid and invalid condition. This result is also seen for the 300 ms SOA, though again only when uncorrected, corrected results appear to show a facilitation effect emerging, which is also seen for the 500 ms SOA where reaction times are faster in the valid compared to both the neutral and invalid conditions, with no clear difference between the neutral and invalid conditions. It is possible that the effects at the short SOAs reflect motor responses, specifically a combination of response priming, causing a facilitation effect (Hommel, 1993, 2011) and cue target conflict, causing an inhibition effect (Green et al., 2013). The clear facilitation without inhibition effect at 500 ms provides further evidence for this conclusion because cue target conflict is found to occur only at shorter SOAs (Green et al., 2013). Therefore, it is possible that this later effect reflects an attention based facilitation effect as it occurs during conscious processing, thought to occur at SOAs upwards of 300 ms (Müller & Rabbitt, 1989).

In this localisation task the responses (left/ right keyboard arrows) were mapped to target location, thus the motor effects of the cue were likely exaggerated due to the Simon effect, which refers to a speeding of responses at the same side that a stimulus is presented. Further, localisation tasks require little, if any, target identity processing and it has been found in previous research that changing the levels of target processing can modulate cuing effects (Bonmassar et al., 2019; Gregory & Jackson, 2021). Therefore, in Experiment 2 a target discrimination task is used. Here responses do not correspond to location and the task requires target processing.

## Experiment 2. discrimination

This study was preregistered on the OSF: https://osf.io/qh7tx, however, a couple of basic changes have been made to the registered protocol. It was said that *t* tests would be one tailed only due to the specificity of the prediction, however, because Experiment 1 showed some effects that went in the other direction it was felt that it would be more transparent to conduct two tailed tests. Further, the registration stated that accuracy outliers would be determined, however, accuracy was high and so this was deemed unnecessary.

## Method

### Participants and apparatus

61 participants (31 females, 30 males, mean age 24 years, range 18–44 years) were recruited through Prolific for payment. Experiment software used matched Experiment 1.

### Stimuli

The avatar cue, stick cue and environment matched those used in Experiment 1. Here the targets were a teapot and a cup, with the teapot created in the same way as described for the cup in Experiment 1.

### Design and procedure

Experiment 2 replicated the design of Experiment 1 using a discrimination task in place of the localisation task. Here participants had to discriminate between a cup and a teapot as quickly and as accurately as possible using the UP arrow on their keyboard if the target was a cup and the DOWN arrow if the target was a teapot. Again, no specific instructions were given about which fingers or which hand to use and the target and the cue remained on screen until a response was made. All other aspects of the procedure are as seen in Experiment 1. The study and materials can be downloaded here: https://osf.io/sra7b/.

## Results

Everyone performed at or above 93% accuracy (median = 98%) and so all participants were retained. Data from incorrect trials were excluded from the reaction time analysis (2% of data).

A repeated measures ANOVA (multivariate) with cue type (avatar, stick), validity (valid, invalid, neutral) and SOA (150, 300, 500, 1000) as within subject factors was conducted on the median reaction times data. This showed a non-significant main effect of cue type, *F*(1, 60) = 0.513, *p* = 0.476, *ηp*^2^ = 0.008, meaning that reaction times were not statistically different for the avatar (*M* = 647 ms) and the stick cue (*M* = 651 ms).

Importantly, there was a significant main effect of validity, *F*(2, 59) = 11.408, *p* < 0.001, *ηp*^2^ = 0.279. Reaction times were significantly faster when the target was validly cued (641 ms) as compared to invalidly cued (652 ms), *t*(60) = − 4.599, *p* < 0.001, Cohen’s *d* = − 0.589 (Bonferroni corrected), further reaction times were significantly faster when the target was validly cued as compared to when the cue stayed central (neutral, 649 ms) *t*(60) = − 3.740, *p* < 0.001, Cohen’s *d* = − 0.479 (Bonferroni corrected) and finally, there was no significant difference between reaction times when the target was invalidly cued as compared to the neutral condition *t*(60) = 0.859, *p* = 1, Cohen’s *d* = 0.110 (Bonferroni corrected).

This validity main effect was not modulated by cue type with no significant interaction between cue type and validity, *F*(2, 59) = 0.661, *p* = 0.520, *ηp*^2^ = 0.022, however, it was modulated by SOA, with a significant interaction between SOA and validity, *F*(6, 55) = 3.738, *p* = 0.003, *ηp*^2^ = 0.290. In direct contrast to the localisation task in Experiment 1, stronger cuing effects were found at the longer SOAs, than the shorter ones, see Table 2 for a full breakdown of the effects. At the 150 ms SOA there was a non-significant difference between reaction times in the valid vs invalid condition and between the valid vs neutral conditions (Bonferroni corrected ps = 1). At the 300 ms SOA there was a non-significant difference between reaction times for the valid vs invalid conditions (Bonferroni corrected *p* = 0.312), but a significant effect when comparing the valid condition to the neutral condition (Bonferroni corrected *p* = 0.012). For the 500 ms and 1000 ms conditions there were significant differences in reaction times between the valid and invalid conditions, and the valid and neutral condition, (Bonferroni corrected ps < 0.05). For all SOAs there were no significant differences between the neutral and invalid conditions, (Bonferroni corrected ps = 1).

**Table 2:** Median RTs (ms) and paired sample *t* test results (*t* value, *p* value) for the cues combined, and each cue at each SOA for the discrimination task (*df* = 60), all *p* values are uncorrected for multiple comparisons and are provided for reference only, asterisks denote those that would remain significant if corrections were made

Cue	SOA	RT valid (SD)	RT invalid (SD)	RT neutral (SD)	Valid vs invalid *t* (*p*)	Valid vs neutral *t* (*p*)	Neutral vs invalid *t* (*p*)
Collapsed across cues	150	677 (84)	676 (85)	672 (88)	0.266 (0.791)	1.21 (0.233)	− 0.89 (0.375)
	300	650 (78)	660 (78)	661 (82)	− 2.28 (0.26)	− 3.41 (0.001)*	0.51 (0.613)
	500	635 (76)	649 (73)	646 (84)	− 4.14 (< 0.001)*	− 3.02 (0.004)*	− 0.50 (0.622)
	1000	613 (65)	628 (80)	625 (76)	− 3.45 (0.001)*	− 3.15 (0.003)*	− 0.88 (0.382)
Avatar	150	667 (87)	669 (87)	666 (89)	− 0.40 (0.694)	0.28 (0.783)	− 0.62 (0.538)
	300	647 (91)	660 (89)	658 (90)	− 2.09 (0.041)	− 2.23 (0.029)	− 0.39 (0.7)
	500	637 (87)	648 (77)	646 (91)	− 1.96 (0.055)	− 1.52 (0.134)	− 0.34 (0.735)
	1000	611 (63)	632 (85)	628 (87)	− 3.70 (< 0.001)*	− 2.80 (0.007)	− 0.90 (0.370)
Stick	150	687 (93)	682 (94)	679 (97)	0.73 (0.469)	1.314 (0.194)	− 0.56 (0.578)
	300	653 (76)	659 (80)	665 (83)	− 1.29 (0.2)	− 2.24 (0.029)	1.04 (0.301)
	500	632 (79)	650 (77)	647 (86)	− 4.17 (< 0.001)*	− 2.80 (0.007)	− 0.48 (0.636)
	1000	615 (78)	624 (82)	623 (74)	− 1.724 (0.090)	− 1.53 (0.131)	− 0.29 (0.772)

There was a significant main effect of SOA *F*(3, 58) = 60.092, *p* < 0.001, *ηp*^2^ = 0.757. There was also a significant interaction between cue type and SOA, *F*(3, 58) = 5.141, *p* = 0.003, *ηp*^2^ = 0.210. Again, comparing like for like (i.e. stick cue at the 150 ms SOA with the gaze cue at the 150 ms SOA) no comparisons are significantly different (all ps > 0.374, Bonferroni corrected). Finally, there was no significant interaction between cue type, validity and SOA, *F*(6, 55) = 0.635, *p* = 0.701, *ηp*^2^ = 0.065.

### Order and gender effects

Cue order was randomised, 30 participants saw the gaze cue first, while 31 saw the stick cue first. There was no main effect of cue order (*p* = 0.966) however, there was an interaction between cue type and cue order, *F*(1, 59) = 11.506, *p* = 0.001, *ηp*^2^ = 0.163. If the stick was presented first, responses were overall faster in the avatar (*M* = 639) than in the stick (*M* = 659) condition (*F*(1, 30) = 10.870, *p* = 0.003, *ηp*^2^ = 0.266). If the avatar cue was presented first, there was no difference between conditions (*p* = 0.10). There was also an interaction between SOA, validity, and cue order *F*(6, 54) = 2.552, *p* = 0.030, *ηp*^2^ = 0.221. If the avatar cue was presented first, there was no interaction between SOA and validity (*p* = 0.188), whereas, if the stick cue was presented first there was an interaction (*F*(6, 25) = 4.879, *p* = 0.002, *ηp*^2^ = 0.539). This possibly drives the effects of this interaction in the main analysis. There were no other significant interactions, (*p* ≥ 0.463).

There was also no main effect of participant gender (*p* = 0.499), however, there was an interaction between participant gender, SOA and validity *F*(6, 54) = 2.324, *p* = 0.045, *ηp*^2^ = 0.205. Exploration of this result shows some differences in the size of the cues effects on attention but no differences in their direction, the results vary in terms of which gender shows stronger effects, with this varying by SOA. There were no other interactions, (ps ≥ 0.324). This indicates that while gender differences in the strength of the cuing effect may be present, they do not appear to change interpretation of the findings overall.

Between studies analysis comparing the overall reaction times in the localisation task (E1) to the discrimination task (E2) showed longer RTs in the discrimination task (649 ms) than the localisation task 431 ms), implying that participants indeed found the discrimination task more difficult, *F*(1, 118) = 138.827, *p* < 0.001, *ηp*^2^ = *0.5*41.

## Summary of results

In Experiment 2 the influence of the cues was tested in a more complex discrimination task. In contrast to Experiment 1, here there was no influence of the cues on attention at the shortest 150 ms SOA, and no evidence of facilitation with inhibition at any SOA. Instead, the evidence suggests that the influence of the cues on attention in this discrimination task is an attentional facilitation effect showing speeded response to validly cued items compared to both invalidly cued items and those in the neutral condition. In contrast to Experiment 1 this included a positive effect of the cues at the longest 1000 ms SOA, with this being driven by the social cue. For the non-social cue at 1000 ms there was no facilitatory effect on attention, but there was also no inhibition of return.

## General discussion

The aim of the presented experiments was to investigate facilitatory versus inhibitory effects of social and non-social cues on target response. Unlike previous investigations of these effects, here the gaze cue was a dynamic realistic avatar, which engaged eye contact prior to the gaze shift, including a dynamic shift in the neutral condition. The control cue was a stick which engaged the participant in a similar manner. The cues both offered realistic motion, shifting over a period of 500 ms which allowed cuing effects to be investigated both during (150 ms, 300 ms) and at completion (500 ms and 1000 ms) of the cues motion.

The role of task in the facilitatory versus inhibitory effects of cuing was investigated using a simple localisation task and a more difficult discrimination task. This was important because localisation tasks and discrimination tasks can reveal different aspects of the cues influence on response times. Responses in localisation tasks directly map onto the target location, therefore demonstrating motor effects of cuing, enhancing response priming (where the participant is primed to respond to a target in the location cued—causing facilitation effects) and cue target conflict effects (where response to an incongruent target is slowed causing inhibition effects). Responses in discrimination tasks are less susceptible to these effects because the response is dependent upon the target identity, not its presence/location.

The cues presented shifted over a period of 500 ms and so the presence of cuing effects at the 150 ms and 300 ms SOAs demonstrate that participant response is affected by the cue during movement. This was most clear for the localisation task, where evidence for facilitation with inhibition was seen at the 150 ms and 300 ms SOAs. For the discrimination task, facilitatory cuing was present at the 300 ms SOA and there were no effects at the shortest 150 ms SOA. It is, therefore, likely that these early effects in the localisation task reflect a combination of response priming (Hommel, 1993, 2011) and cue target conflict (Green et al., 2013) possibly due to an anticipatory object tracking effect (Hayhoe et al., 2012) whereby the participant anticipates the nature of the cue shift and readies their response for that location. This replicates the results of Hietanen (1999) who also found facilitation with inhibition at short SOAs using a localisation task, and offered a similar explanation of the results, explaining that the cue primes a motor programme which causes a speeded response when the target is congruent, i.e. response priming, causing the facilitation effect (e.g. Hommel, 1993, 2011) and a slowed response due to needing to ‘reprogramme’ when the target is incongruent, i.e. a cue target conflict, causing the inhibition effect (e.g. Green et al., 2013). For the discrimination task it is possible that at the shortest SOA the cues movement attracted attention (i.e. Kawahara et al., 2012) which disrupted the target processing required for this more complex task, with this being overcome at the 300 ms SOA by conscious attentional processes (Müller & Rabbitt, 1989).

The presence of facilitatory cuing at the 500 ms SOA in the localisation task, and in all but the 150 ms SOA in the discrimination task, reflects a general facilitation effect of the cues. Reaction times are faster in the valid condition than both the neutral and invalid conditions, with no difference between the neutral and invalid conditions. While this response pattern is considered to reflect a general priming of a response to the cued location, occurring without attention (Hommel, 1993, 2011), such conclusions are based upon the use of Simon style left and right lateralised tasks. Here the discrimination task had no such response mapping, indicating the involvement of attention, further the presence of this effect at the longer SOAs (upwards of 300 ms) means that it is likely that conscious processing was involved (Müller & Rabbitt, 1989). Previous research using a discrimination task (Friesen & Kingstone, 1998) has also provided evidence for facilitation without inhibition, with these results occurring most clearly at 300 ms and 600 ms SOA. It is therefore likely that orienting effects at the longer SOAs in both the discrimination and localisation tasks are driven by a conscious attention shift to the cued location driven by the cue, i.e., attentional facilitation. This is supported by research that shows that the effects of neutral-expression gaze cues on attention are disrupted by the performance of concurrent cognitive tasks that deplete attentional resources (Bobak & Langton, 2015; Chen et al., 2021; Pecchinenda & Petrucci, 2016), indicating that attention is indeed required to follow the gaze cue. However, note that in other studies concurrent cognitive tasks have not impacted the cuing effect (Hayward & Ristic, 2013; Law et al., 2010; Xu et al., 2011), though it has been argued that these studies did not sufficiently deplete attentional resources (Bobak & Langton, 2015). The research presented here therefore adds further evidence to the idea that central cues, including gaze cues and here the non-social stick cue, influence attention in a facilitatory way, causing enhancement of the cued location without causing inhibition of the un-cued location at longer, volitional SOAs. These results demonstrate the importance of task when investigating these effects.

Here, both the social and non social cue engaged with the participant prior to gaze shift by moving from looking or pointing at the table to looking or pointing at the participant. For the gaze cue this meant that the participant was engaged in eye contact prior to gaze shift. This is a key aspect of social behaviour (Emery, 2000; Kleinke, 1986) which engages and modulates distinct social processes (Capozzi & Ristic, 2018; Conty et al., 2016; Senju & Johnson, 2009) including the gaze cuing effect (Bristow et al., 2007; Xu et al., 2018). Importantly, here it was demonstrated that it is possible to assess facilitation versus inhibition effects while engaging eye contact by using a dynamic neutral cue. The results of the reported studies show that this method produces results that are comparable in nature to those seen previously where the neutral cue showed no gaze behaviour. This is important for social cuing studies going forwards as the use of eye contact and neutral cues are both important factors in further understanding of the effects of gaze cues, in particular in terms of their effects on higher cognition, for example on memory (Dodd et al., 2012; Gregory & Jackson, 2017) and on object appraisal (Bayliss et al., 2006).

Realism was an important aspect of the cues presented, and here the human avatars were presented with their full upper body shown. In addition, targets were presented on a table below the cue, thus adding contextual relevance to the target. This use of a table meant that the targets did not appear directly to the side of the cue. It was therefore possible for participants to complete the task by keeping attention on the table and ignoring the cue. It is clear from the results that the participants did not do this, instead following the cues despite their unhelpful nature. This provides further evidence that these cues are difficult to ignore and indicates that findings can be generalised to a real-world context.

The general absence of effects modulated by cue type is consistent with other more traditional cuing studies, where no statistical difference in cuing magnitude is seen between gaze and arrow cues (e.g. Green et al., 2013; Hietanen et al., 2008; Hommel et al., 2001; Ristic et al., 2002; Tipples, 2002; Xu & Tanaka, 2015). Generally, differences are more likely to be seen with higher order effects on memory and object appraisal, where gaze cues show an effect and non-social arrow cues do not (Bayliss et al., 2006; Dodd et al., 2012; Gregory & Jackson, 2017). This stick cue is therefore an important alternative to the traditional arrow cue and would be highly useful in research conducted in young children and infants. This is because while gaze cuing occurs from as young as 3 months (Hood et al., 1998), response to arrow signals takes longer to emerge. Research shows that children can follow arrow cues when told to from around 30 months, but do not show adult like orienting to the symbolic aspects of the cues until the age of 5 (Jakobsen et al., 2013). Therefore, this stick cue could be highly useful in studies of the gaze cuing effect in younger children.

Nevertheless, despite the general similarity in effects, there were some key differences between the social and non-social cue seen which are worth commenting upon. In the localisation task at the 1000 ms SOA the social avatar cue showed almost no effect on attention while the non-social stick cue showed evidence of inhibition of return (Klein, 2000). Inhibition of return occurs after around 200–300 ms in peripheral cuing tasks, characterised by faster response to the uncued location. While this effect is well-documented in peripheral cuing studies it tends to occur much later in central cuing studies, both for social and non-social cues (Frischen & Tipper, 2004; Frischen et al., 2007a, 2007b; Weger et al., 2008). This effect was not replicated in Experiment 2, where for the avatar cue a facilitation effect persisted, while for the stick cue the effect was no longer significant but was in the same direction as that of the social cue. It is possible that for the localisation task, the primed motor response to the stick cue is eventually inhibited, causing the slowed motor response to the previously cued location (Taylor & Klein, 2000), with this taking longer than is seen in peripheral cuing studies due to the central nature of the task. This inhibition effect may also act upon the avatar cue, however, while the primed motor response may be inhibited, the response to the gaze direction may remain due to the powerful nature of the gaze cuing effect (Frischen et al., 2007a, 2007b; Pesimena et al., 2019), thus countering the inhibited motor response results in a weak or absent effect of the cue. Indeed, inhibition of return is rarely found for social gaze cues (McKee et al., 2007).

A key aim of this study was to create a useful neutral cue for investigating the gaze cuing effect while allowing for eye contact and natural head movement. While results seen reflect those of previous work, validating the neutral cue used, it is important to note that it is difficult to create a true neutral condition from which to compare effects (Jonides & Mack, 1984). Here, all three cuing conditions involved motion, shifting from pointing at the participant to down in the neutral condition or left/right in the valid and invalid conditions. Therefore, it is possible that this motion captured attention away from the target location in a similar way for both the neutral and invalid conditions. However, this is also a likely scenario in neutral conditions used previously as the direct gaze used is known to capture and hold attention (Senju & Hasegawa, 2005; Senju & Johnson, 2009). Importantly, for the neutral cue presented here the motion directed the participants attention towards the table where the items subsequently appear.

In conclusion the research presented demonstrates facilitatory effects on attention can be found using dynamic social avatars and dynamic non-social cues. Further, by using localisation and discrimination tasks it was possible to demonstrate attentional compared to motor-based effects of the cues. The use of dynamic realistic stimuli allowed a better understanding of how attention might be influenced in more realistic scenarios. These sophisticated social and non-social cues are therefore well placed for research investigating more complex higher order effects of cues on cognition.

## Declarations

### Availability of data and material

All data and materials are available on the Open Science Framework (OSF): https://osf.io/5mz9j/.

### Code availability

Code related to the study is available on the OSF, including the programmed study and data extraction scripts: https://osf.io/5mz9j/.
